# Cytotoxic and targeted therapy for hereditary cancers

**DOI:** 10.1186/s13053-016-0057-2

**Published:** 2016-08-23

**Authors:** Aglaya G. Iyevleva, Evgeny N. Imyanitov

**Affiliations:** 1 N.N. Petrov Institute of Oncology, Pesochny-2, St. Petersburg, 197758 Russia; 2 St. Petersburg Pediatric Medical University, St. Petersburg, 194100 Russia; 3 I.I. Mechnikov North-Western Medical University, St. Petersburg, 191015 Russia; 4 St. Petersburg State University, St. Petersburg, 199034 Russia

**Keywords:** Hereditary cancer syndromes, Familial cancer, Breast cancer, Ovarian cancer, Colorectal cancer, Cytotoxic therapy, Targeted therapy, Predictive markers, BRCA1, BRCA2

## Abstract

There is a number of drugs demonstrating specific activity towards hereditary cancers. For example, tumors in BRCA1/2 mutation carriers usually arise via somatic inactivation of the remaining BRCA allele, which makes them particularly sensitive to platinum-based drugs, PARP inhibitors (PARPi), mitomycin C, liposomal doxorubicin, etc. There are several molecular assays for BRCA-ness, which permit to reveal BRCA-like phenocopies among sporadic tumors and thus extend clinical indications for the use of BRCA-specific therapies. Retrospective data on high-dose chemotherapy deserve consideration given some unexpected instances of cure from metastatic disease among BRCA1/2-mutated patients. Hereditary non-polyposis colorectal cancer (HNPCC) is characterized by high-level microsatellite instability (MSI-H), increased antigenicity and elevated expression of immunosuppressive molecules. Recent clinical trial demonstrated tumor responses in HNPCC patients treated by the immune checkpoint inhibitor pembrolizumab. There are successful clinical trials on the use of novel targeted agents for the treatment or rare cancer syndromes, e.g. RET inhibitors for hereditary medullary thyroid cancer, mTOR inhibitors for tumors arising in patients with tuberous sclerosis (TSC), and SMO inhibitors for basal-cell nevus syndrome. Germ-line mutation tests will be increasingly used in the future for the choice of the optimal therapy, therefore turnaround time for these laboratory procedures needs to be significantly reduced to ensure proper treatment planning.

## Background

First tumor-predisposing germ-line mutations were discovered a quarter of century ago and were immediately translated into appropriate diagnostic tests [1–5]. Identification of mutation carriers among cancer patients, their yet healthy relatives and, to a lesser extent, some other individuals rapidly entered clinical routine and saved thousands of lives by delivering specific diagnostic and preventive efforts to the subjects at-risk. However, treatment schemes for hereditary and sporadic cancers remained virtually identical until this decade, therefore the genetic testing was usually considered rather as a part of the follow-up than the component of the initial decision-making process. We are currently witnessing a cultural change in clinical perception of hereditary cancers. It is getting increasingly recognized that many germ-line mutation-driven tumors develop via authentic molecular pathways and therefore have a unique spectrum of sensitivity to both conventional cytotoxic compounds and novel targeted drugs [6–9]. Many doctors now request rapid genetic testing at the time of treatment planning, and these attitudes are likely to become mandatory for the good clinical practice in a very near future. Here we review recent advances and controversies in the therapy of hereditary cancers (Table 1).

**Table 1:** Examples of cytotoxic and targeted drugs showing promising activity towards hereditary cancers

Hereditary cancer type	Drug
BRCA1/2-driven cancers (breast, ovarian, prostate, pancreatic, stomach, etc.)	Genotoxic agents: platinum compounds, PARP inhibitors, mitomycin C, pegylated doxorubicin, etc.; high dose chemotherapy
Hereditary non-polyposis colorectal cancer	Immune checkpoint inhibitors: pembrolizumab
Familial adenomatous polyposis	Non-steroidal anti-inflammatory drugs (sulindac) and EGFR inhibitors (erlotinib)
Tumors arising in patients with tuberous sclerosis (giant-cell astrocytomas, angiomyolipomas)	mTOR inhibitors: everolimus
Tumors associated with the basal-cell nevus syndrome (basal-cell carcinomas, keratocystic odontogenic tumors)	SMO inhibitors (vismodegib), COX2 inhibitors (celecoxib), antifungal drugs with Hedgehog pathway inhibitory activity (itraconazole)
Hereditary medullary thyroid cancer	RET inhibitors (vandetanib, cabozantinib)

Note: See the text for comments and references

### BRCA1 and BRCA2

#### Therapeutic window in BRCA-driven tumors

**Fig. 1:**
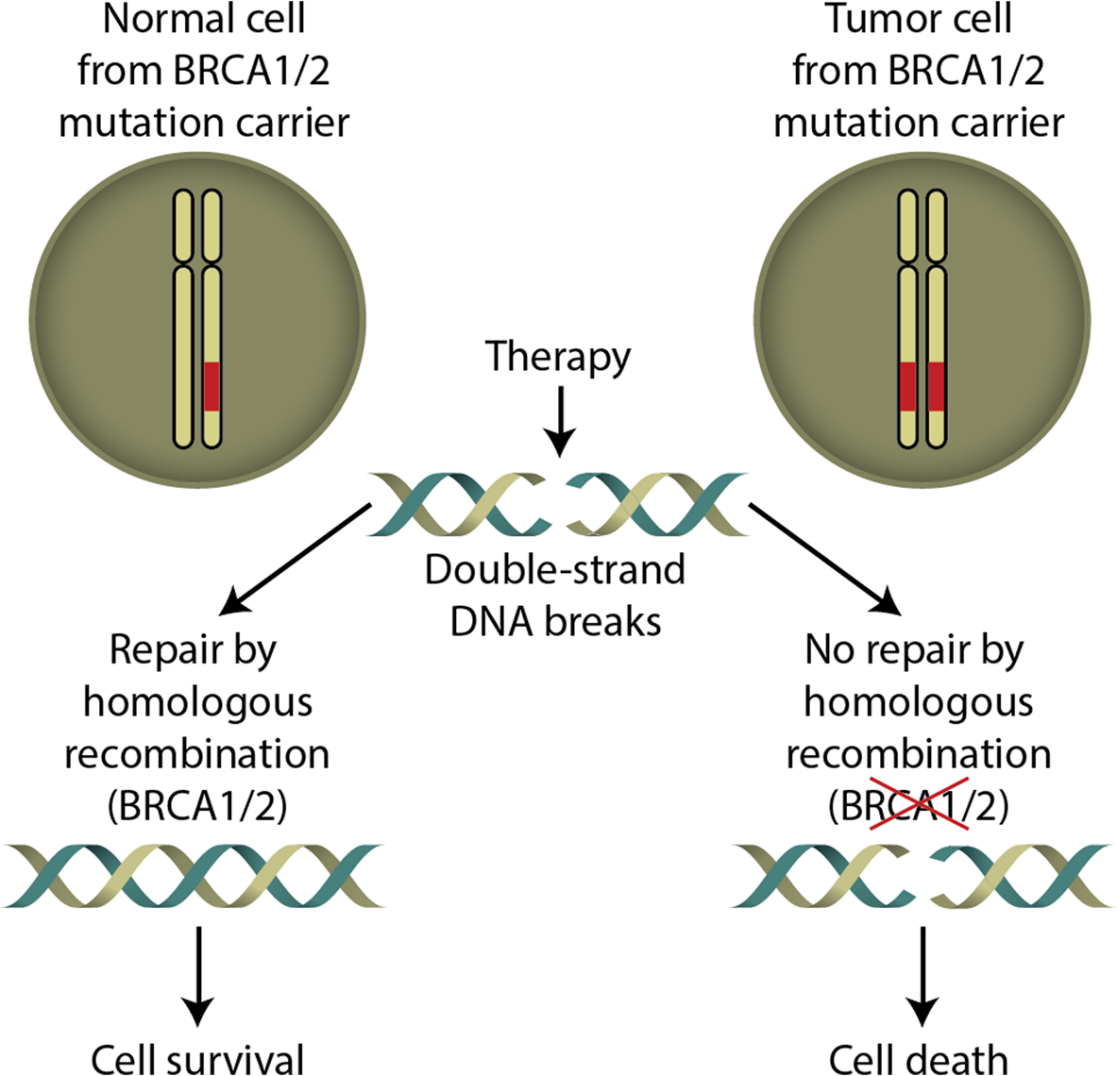
Selective sensitivity of BRCA1/2-associated tumors to genotoxic agents. Normal cells from BRCA1/2 mutation carriers retain full capacity of genome maintenance mechanisms (left). Development of tumors in these patients involves somatic inactivation of the remaining BRCA1/2 allele, therefore malignant cells are unable to cope with double-strand DNA breaks (right)

Breast-ovarian hereditary cancer syndrome is by far more common than other categories of familial cancers, with BRCA1 and BRCA2 being among the most studied genes. BRCA1/2-driven tumors usually arise via 2-hit mechanism: while the involved gene is present in heterozygous but still proficient state in the normal cells of the carrier, cancer cells are characterized by somatic loss of the remaining BRCA allele and therefore demonstrate deficiency in DNA repair by homologous recombination (HR). This opens an elegant therapeutic window by making tumor cells specifically vulnerable to DNA damaging drugs and poly(ADP-ribose) polymerase (PARP) inhibitors (Fig. 1). For example, platinating drugs induce DNA crosslinks, which cannot be effectively repaired in the absence of HR. Similarly, PARP inhibition results in accumulation of single-strand DNA breaks, which are subsequently converted to double-strand DNA breaks and turn out to be lethal for BRCA-deficient cells. This concept was initially confirmed in various laboratory studies and recently received validation in a series of clinical investigations [10–13].

#### Platinum-based therapies

High sensitivity of BRCA1-driven tumors to cisplatin was initially demonstrated in a small Polish neoadjuvant breast cancer (BC) study involving mainly patients with small tumors [14]. Given that the pathologic complete response (pCR) was observed in 9 (90%) out of 10 included women [14], cisplatin quickly became a popular therapeutic option for hereditary BC in Poland. Recently Byrski et al. [15] reported an update on their experience of using cisplatin in the neoadjuvant BC setting: pCR was documented in 65/107 (61%) patients. Cisplatin appears to be clearly superior when compared to other schemes of preoperative therapy in BRCA1 mutation carriers [16, 17]. Furthermore, remarkable performance of cisplatin in chemonaive BRCA1-related BC was confirmed in several independent reports [18–20]. The key question is whether these high pCR rates will indeed translate into improved long-term outcomes; pooled analysis of neoadjuvant BC trials provides encouraging information suggesting that the relationship between pCR and survival is particularly strong in patients with the triple-negative BC disease [21].

Data on the use of platinum compounds in metastatic BC are limited. Byrski et al. [22] recruited both chemonaive and pretreated BRCA1 mutation carriers for cisplatin study, and reported the response in 16/20 (80%) cases with median progression-free survival (PFS) 12 months. Isakoff et al. [23] used either cisplatin or carboplatin in 11 patients with BRCA1/2-driven BC, most of whom experienced prior chemotherapy; the response was seen in 6 (54.5%) women, however its duration was short (3.3 months). Tutt et al. [24] compared carboplatin vs. docetaxel in triple-negative metastatic BC; for BRCA1/2 mutation carriers, the response rates were 68% vs. 33%, and PFS was 6.8 months vs. 3.1 months. There are several factors which critically compromise the assessment of platinum compounds in women with metastatic hereditary BC. First, the majority of patients presenting with inoperable disease have a history of prior chemotherapy; this is indeed an important confounding factor for BRCA-driven cancers, given that the development of the drug resistance frequently involves restoration of BRCA function [25]. Second, cisplatin and carboplatin have distinct efficacy and therefore have to be considered separately [26]. Third, there are multiple ongoing trials on PARP inhibitors (PARPi); it is probable that the cohort of patients receiving platinum therapy is somehow enriched by women, who were not included in PARPi studies due to contraindications or some other reasons.

Platinum-based therapy forms a backbone for the standard systemic treatment of ovarian cancer (OC). Multiple studies indicate that BRCA1/2-driven OC are characterized by increased sensitivity to platinating agents as compared to sporadic carcinomas [27–30]. Interstudy comparison of long-term results of the OC therapy is complicated, because OC outcomes are significantly influenced by the quality of cytoreductive surgery [31].

#### PARP inhibitors

There is a number of PARP inhibitors, including olaparib (Lynparza, AstraZeneca), veliparib (ABT-888, AbbVie), rucaparib (AG-014699, Clovis Oncology), niraparib (MK-4827, Tesaro), talazoparib (BMN-673, BioMarin Pharmaceutical), etc. [13]. Iniparib (Sanofi) was also initially developed as a PARP inhibitor; however recent studies showed that it has limited if any PARP-inhibiting activity and therefore cannot be considered as a drug belonging to PARPi class [32, 33].

Olaparib is the only PARP inhibitor already approved for the clinical use. Its initial trial as monotherapy agent demonstrated its preferential effect towards cancers arising in BRCA1/2 germ-line mutation carriers [34]. Subsequent study involving chemotherapy-pretreated ovarian cancer patients revealed that the efficacy of olaparib is more pronounced in women with platinum-sensitive disease and prolonged platinum-free interval [35]. Overall, there is consistent evidence for activity of olaparib in BRCA1/2-related cancers [36–41]. Olaparib received accelerated approval from the Food and Drug Administration (FDA) for the use in patients with BRCA1/2-driven ovarian cancers who received at least 3 prior lines of chemotherapy. In addition, this compound was assessed as a maintenance treatment in OC patients experiencing response to platinum-based therapy; it prolonged PFS as compared to placebo (11.2 vs. 4.3 months) in cases with germ-line or somatic BRCA1/2 mutations, although overall survival did not differ between these 2 arms [42]. Based on these data, olaparib is now approved for OC maintenance therapy by the European Medicines Agency (EMEA).

Given that neither olaparib nor conventional cytotoxic compounds are capable to achieve high rate of prolonged complete responses while administered separately, there is some interest to the use of these drugs in combination. Preclinical experiments support this concept [43–45]. Combination of olaparib and carboplatin has already been assessed in a Phase 1/1b study involving mixed population of patients with BRCA1/2 germ-line mutations, however only 1 out of 42 evaluable patients achieved complete response [46]. Another Phase I study demonstrated reasonable tolerability of combining olaparib with pegylated liposomal doxorubicin [47]. Oza et al. [48] studied OC patients with recurrent platinum-sensitive disease; while analyzing the subgroup of BRCA1/2 germ-line mutation carriers, they revealed that addition of olaparib to paclitaxel and carboplatin improved PFS as compared to chemotherapy alone.

Only a few studies assessed the efficacy of olaparib against standard cytotoxic treatment. Comparison of olaparib vs. pegylated liposomal doxorubicin (PLD) in recurrent BRCA1/2-associated ovarian cancer produced similar results for both drugs [49]. It has to be mentioned that PLD showed noticeably higher activity in BRCA1/2 mutation carriers as compared to historical non-selected OC series [50].

Data on the use of other PARP inhibitors are less extensive. Veliparib was evaluated in heavily pretreated ovarian cancer patients with BRCA1/2 mutation, and induced tumor responses in 13/50 (26%) women; similarly to experience with olaparib, there was a clear difference in response rates between platinum-resistant and platinum-sensitive disease (20% vs. 35%) [51]. Niraparib was assessed in a Phase I study; it demonstrated responses in 8/20 (40%) ovarian and 2/4 (50%) breast BRCA1/2-related cancers [52]. High disease control rates were also reported in a rucaparib monotherapy trial [53].

It is important to acknowledge, that all published clinical trials on PARP inhibitors involved pretreated BRCA1/2-mutated patients. This could be a critical limitation, as at least some BRCA1/2-driven tumors demonstrate restoration of intratumoral BRCA1/2 function during cytotoxic therapy [25, 29]. Low efficacy of PARPi in platinum-resistant as compared to platinum-sensitive ovarian cancer supports this assumption [51].

#### Other BRCA-specific therapies

In addition to cisplatin, there are several other non-expensive cytotoxic drugs showing BRCA-specific activity in preclinical experiments [6]. Based on these data, Moiseyenko et al. [54] administered single-agent mitomycin C (10 mg/m2, every 4 weeks) to 12 heavily pretreated ovarian cancer patients and obtained encouraging results: there was 1 complete response, 2 partial responses and 6 instances of the disease stabilization.

Trabectedin (Yondelis, Janssen) is a novel DNA damaging cytotoxic drug approved by FDA and EMEA for the therapy of inoperable soft tissue sarcomas [55]. In addition, it is used in some countries for the treatment of relapsed ovarian cancer in combination with pegylated liposomal doxorubicin [56]. It was assessed as a monotherapy in pretreated metastatic BRCA1/2-mutated breast cancer patients: 6/35 (17%) evaluable women experienced response, and median PFS approached to 3.9 months [57]. Promising activity of trabectedin was also shown in chemotherapy-pretreated hereditary ovarian cancer patients, although similar rates of tumor responses were observed in BRCA1/2 mutation carriers vs. non-carriers [58].

Eribulin (Halaven, Eisai) is a novel microtubule inhibitor, which demonstrated improvement of overall survival in patients with metastatic breast cancer after failure of multiple lines of systemic therapy. It was evaluated in combination with carboplatin in neoadjuvant trial involving triple-negative breast cancer patients. The study included 3 patients with BRCA1/2 mutation; clinical response was observed in all these women, with 2 of them achieving pathologic complete response [59].

BRCA1 is required for the execution of taxane-induced apoptosis, and at least some data indicate that taxane-containing regimens show limited efficacy towards BRCA1-associated breast cancers [16, 17, 60]. It is essential to acknowledge that some breast cancer studies do not support this concept [61]. Furthermore, paclitaxel monotherapy is effective in relapsed ovarian cancer in BRCA1 mutation carriers [62], although this study did not consider possible restoration of BRCA1 function during the prior therapy [25]. Recently Burness et al. [63] communicated 2 cases of BRCA1-associated chemonaive breast cancers, which demonstrated complete response to the paclitaxel monotherapy. These data deserve high level of attention, as they clearly contradict to the current views on the mechanisms of taxane action [7]. It is almost certain, that some aspects of cytotoxic effects of taxanes still remain unrecognized, however at least 2 reservations need to be kept in mind with regard to the report of Burness et al. [63]. First, there could be a publication bias, i.e. unexpected observations have significantly better chances to be published than routine clinical experience. Second, somatic inactivation of the wild-type BRCA1 allele may not be the only mechanism of breast cancer development in BRCA1 mutation carriers, as at least a subset of BRCA1-driven tumors appear to retain BRCA1 function [64, 65]; it is tempting to speculate that the sensitivity to taxanes is preserved in the latter category of BC.

Anthracyclines appear to exert substantial activity against BRCA1/2-driven tumors [6, 16, 17], with the novel formulations of these drugs producing remarkable responses in a subset of patients [49, 66–68]. There are limited data on the use of alkylating cytotoxic drugs in BRCA1/2-mutated cancers [7]. Kummar et al. [69] recently investigated low-dose daily cyclophosphamide in pretreated ovarian cancer patients, and observed 1 complete and 6 partial responses among 38 treated women.

There are some data on the activation of PTEN/PI3K/AKT/mTOR pathway in cancers arising in BRCA mutation carriers. Two mTOR inhibitors, temsirolimus and everolimus, are already available for the clinical use, however their performance in BRCA-driven cancers has not been assessed yet in preclinical or clinical settings. Recent studies demonstrate that mTOR down-regulation may sensitize cancer cells to PARP inhibitors [70, 71]. There is also some interest to the evaluation of BRCA-specific therapeutic potential of PI3K inhibitors [72–74].

BRCA1/2-deficient tumors are characterized by increased mutational load and therefore appear to be more antigenic than sporadic cancers. In accordance with this, these cancers demonstrate increased lymphocyte infiltration and show distinct pattern of expression of immune-related molecules [75, 76]. These data justify clinical trials involving immune checkpoint inhibitors.

#### Rare types of BRCA-associated cancers

Carriers of BRCA1 and BRCA2 germ-line mutations usually develop breast and/or ovarian cancers, however there is also some association with other cancer types. Inherited BRCA2 heterozygosity is associated with elevated prostate cancer risk. BRCA2-driven prostate cancers demonstrate good response to platinum-containing therapy and PARP inhibitors [38, 77–79]. Similar experience is obtained with pancreatic cancer [6, 9, 38, 80]. It remains under-recognized that some BRCA heterozygotes develop gastric cancer; causal relationship with BRCA status is supported by the evidence for tumor-specific inactivation of the wild-type BRCA allele. BRCA1-related stomach cancers are characterized by prolonged response to platinum containing therapy [81].

#### BRCA-ness

Many sporadic tumors share biological characteristics with BRCA1/2-associated hereditary cancers [82, 83]. For example, tumor-specific somatic inactivation of BRCA1 is the frequent cause of BRCA-ness phenotype. It may be manifested by the loss of BRCA1 expression, caused by either BRCA1 promoter hypermethylation or yet unknown reasons, or by mutational inactivation of both alleles of BRCA1 [84, 85]. Disruption of other genes belonging to HR pathway may also lead to similar consequences [78].

While some “BRCA-ness assays” rely on the identification of genetic causes of this phenotype, i.e. the detection of biallelic inactivation of BRCA or similar genes, novel generation of diagnostic tests utilizes characteristic mutational pattern of BRCA1/2-driven (HR-deficient) tumors [86–89]. For example, chromosomal instability results in a specific tumor karyotype, which can be revealed by array comparative genomic hybridization (aCGH) or some other techniques [90, 91]. BRCA1/2-deficient tumors demonstrate increased number of losses of heterozygosity (LOH) of a certain size (more than 15Mb but less than the entire chromosome) [92]. There is also a correlation between BRCA1/2 deficiency and allelic imbalances at telomeres [93]. Furthermore, BRCA1/2 inactivation manifests by large-scale chromosomal rearrangements [94].

In addition to characteristic mutational signatures, HR-deficient tumors demonstrate specific expression profiles [95, 96]. Cells with functional HR form RAD51 foci after DNA damage; there are attempts to establish ex vivo tumor HR testing using the analysis of induced RAD51 response [97].

Clinical data on the role of BRCA-ness demonstrate high level of consistency. BRCA-ness phenotype is associated with higher tumor sensitivity to platinum-containing agents and PARP inhibitors [23, 42, 78, 98, 99] and poor response to taxane-containing regimens [100]. The discovery of BRCA-ness (HR-ness) in a subset of sporadic tumors significantly extends potential indications for the drugs, which were initially considered to be active only in BRCA1/2-related hereditary cancers [82, 83].

It is essential to acknowledge that the participation of BRCA1 in DNA damage repair is not limited to HR, therefore the definitions of BRCA-ness and HR-ness are not necessarily identical. Hill et al. [101] studied triple-negative breast cancer cell lines and revealed no major HR defects. However, these cells exhibited deficiency in the repair of stalled replication forks, yet another feature of BRCA1 inactivation. Importantly, while the disruption of HR is associated with sensitivity to both cisplatin and PARPi, cells with abnormal repair of stalled replication forks are more selective, i.e. they would respond to the former but not to the latter [101]. These subtle differences may partially explain why there is no complete cross-resistance between DNA damaging cytotoxic drugs and PARP inhibitors [40, 51].

#### Mechanisms of resistance to BRCA-specific therapies

Most of disease-causing BRCA1/2 mutations are represented by relatively small alterations in DNA sequence, which cause frame-shift or generation of premature stop-codon. There is a series of fascinating reports, which demonstrate a somatic restoration of the open reading frame of BRCA genes in the therapy-resistant tumor cells; this is achieved by the occurrence of the second mutation in the proximity to the first one. As expected, second mutations are observed both in platinum- and in PARPi-treated tumors [25, 102–108].

**Fig. 2:**
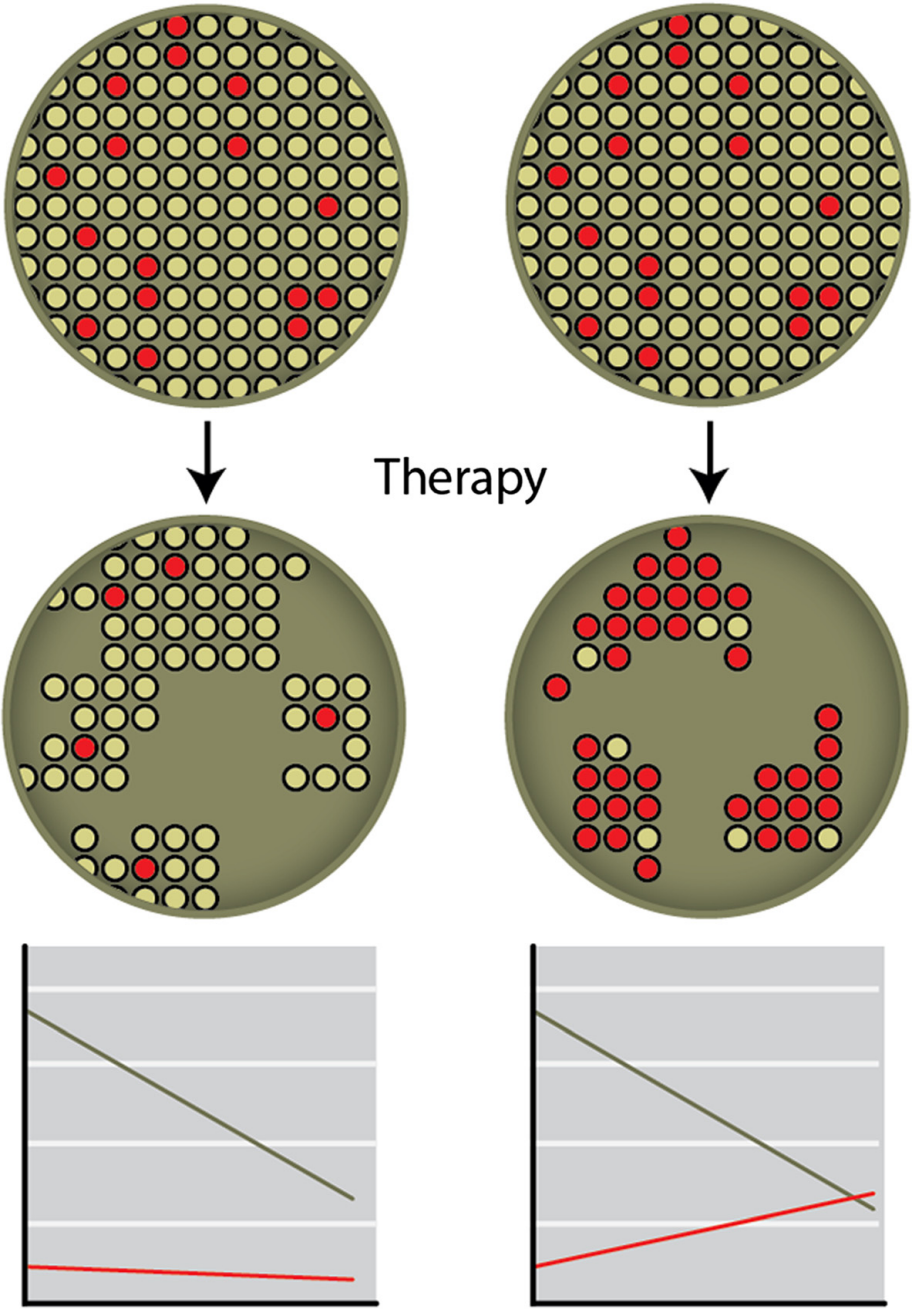
The dynamics of distinct tumor cell populations upon systemic therapy. Many tumors respond well to the initial therapy; although the existence of intratumoral cellular heterogeneity is widely acknowledged, it is generally believed that the evolution of treatment-resistant clones requires additional genetic events and usually takes at least several months (left). Our data indicate that even short-term (neoadjuvant) exposure of BRCA1-driven tumors to platinum therapy results in the replacement of tumor mass by BRCA1-proficient cells [29]. While cells with BRCA1 LOH die almost immediately after beginning of the treatment, clones with retained BRCA1 continue to proliferate during platinum exposure and rapidly repopulate the tumor lump (right)

While the above mechanisms represent mutational evolution of tumor genome under the pressure of BRCA1/2-specific therapy, we observed a distinct root of acquiring platinum resistance [29]. We analyzed somatic BRCA1 status in hereditary ovarian cancers undergoing short-term preoperative therapy (on average, 3 cycles of platinum-containing cocktails given with 21-day intervals). Astonishingly, while the chemonaive carcinomas showed LOH characteristic for BRCA1-associated malignancies, the wild-type allele was preserved in the residual tumor masses removed upon surgery. Given that LOH is not an early event in BRCA1-driven tumorigenesis and that tumors from BRCA1 mutation carriers are known to contain a fraction of cells with the intact wild-type allele [109], this phenomenon can be explained by rapid selection of preexisting BRCA1-proficient cells during platinum exposure. The mere fact of selection of treatment-resistant cells upon the therapy is not at all surprising; what is indeed entirely unexpected, is the speed of this transition. On the level of clinical measuring of the changes in tumor size, the process of acquiring drug resistance by initially chemosensitive tumors usually takes several months; our data indicate that truly responsive neoplastic cells die within very first weeks (days?) of treatment, and they are rapidly replaced by the clones with potentially chemorefractory phenotype (Fig. 2). These data cast some doubt on the ideology of neoadjuvant treatment. Preoperative therapy is commonly viewed as a highly informative in vivo test for the actual tumor sensitivity [110]. If pronounced reduction of tumor burden is observed before the surgery, the same schemes are often administered in the adjuvant setting [111]. However, if the turnover of tumor cell populations is indeed so rapid, and the surgically removed cancer mass is biologically distinct from the initial malignancy, the rationale for continuing the same therapy after the surgery may look questionable.

There are some other proposed mechanisms of the resistance of BRCA1/2-mutated cells to the specific therapy, such as inactivation of TP53BP1 protein, efflux of platinum drugs or PARP inhibitors, or some other molecular events [25, 112–115]. However, they were demonstrated mainly in laboratory models, and their actual clinical relevance remains uncertain.

#### High-dose chemotherapy

High-dose chemotherapy followed by autologous hematopoietic stem cell transplantation is used for the treatment of germ-cell tumors, several hematological malignancies, childhood cancers etc. It was a popular experimental approach for the treatment of breast cancer in mid 1990s, but failed to demonstrate improved long-term outcomes in unselected BC patients [116–118]. However, retrospective analysis of women with metastatic BC revealed several instances of unexpectedly long remission of the disease; importantly, these survivors are enriched by BRCA1/2 germ-line mutation carriers, which is in good agreement with the data on increased chemosensitivity of BRCA-driven BC [119]. Furthermore, patients with BRCA1-like stage III BC show substantial benefit from adjuvant high-dose therapy [91, 120], and this effect correlates with the molecular status of BRCA1-related pathways [115]. There is also a number of case reports describing patients with germ-line BRCA1/2 mutations, who were treated by high-dose chemotherapy for the metastatic disease and remained tumor-free for years [121, 122]. Given that many BRCA1/2 mutation carriers are diagnosed with metastatic cancer at a relatively young age and therefore retain sufficiently good health status to survive a risky intervention, there is a rationale for considering high-dose therapy trials for this uniquely chemosensitive category of tumors.

#### Adverse effects of BRCA-specific therapy in the germ-line mutation carriers: potential impact of haploinsufficiency

Although normal cells in BRCA1/2 mutation carriers retain a wild-type copy of the involved gene and therefore are able to cope with DNA damage, some experiments suggest that the loss of even single BRCA1 allele results in some decrease of BRCA1 functional capacity [123]. If the data on BRCA haploinsufficiency are applicable to the individuals with inherited BRCA defects, one may expect distinct pattern and severity of adverse effects of cancer therapy in the mutations carriers vs. non-carriers. The majority of available clinical investigations did not acknowledge unexpected adverse reactions [6, 22, 119], which may be interpreted, with some caution, in favor of normal tolerability of chemotherapy in BRCA1/2 mutation carriers. Furthermore, systematic single-center analysis of BC patients receiving conventional cytotoxic drugs did not reveal increased toxicity in BRCA1/2 heterozygotes vs. other women [124]. Nevertheless, Moon et al. [125] observed increased incidence of hypersensitivity reactions in BRCA1/2-mutated ovarian cancer patients receiving carboplatin and olaparib. There is also a discussion whether therapeutic radiation is associated with increased risk of induced cancers in BRCA1/2 heterozygotes [126, 127].

### Novel types of hereditary breast cancer

While BRCA1/2-related cancers have been studied with a high level of comprehension, the available information on clinical behavior of novel categories of hereditary breast cancer remains very limited. Importantly, BC arising in CHEK2, NBN/NBS1 and BLM heterozygotes usually demonstrates retention of the wild-type allele in the tumor, therefore there is no ground to expect selective chemosensitivity in these tumor types [128]. In agreement with mechanistic considerations, Chrisanthar et al. [129] observed several instances of resistance of CHEK2-associated BC to epirubicin. Pfeifer et al. [17] described 8 CHEK2 mutation carriers receiving neoadjuvant therapy, with none of them achieving pathologic complete response; 4 out of these 8 women experienced objective clinical response, however the reduction of the tumor size was observed only in 1 out of 4 patients treated by anthracycline-based therapy without taxanes. While data of Chrisanthar et al. [129] and Pfeifer et al. [17] indicate relative chemoresistance of CHEK2-driven BC at least to anthracyclines, a large study of Kriege et al. [130] provided conflicting evidence. The latter group described 62 CHEK2-mutated metastatic BC cases, and the response rates to cytotoxic and endocrine therapy did not differ between CHEK2 heterozygotes and mutation-free controls [130].

### Hereditary colon cancer

There are multiple types of hereditary colon cancer [131], with the so-called hereditary non-polyposis colorectal cancer (HNPCC) syndrome being the most studied type of this disease. HNPCC is caused by germ-line mutations in DNA mismatch repair genes; HNPCC-related tumors are characterized by a unique phenotypic feature, i.e. high-level microsatellite instability (MSI-H). It is beyond the doubt that MSI-H tumors have distinct biological properties. In particular, they have increased level of antigenicity due to accumulation of multiple background mutations, and therefore are characterized by a relatively good prognosis [132–134].

MSI-H carcinomas combine two distinct categories of malignancies. First, there are hereditary cancers obtained from the germ-line mutation carriers; they tend to be associated with younger age of the patients. Second, there are sporadic MSI-H tumors; they often arise in elderly subjects and frequently carry an actionable activating mutation in BRAF oncogene. In addition to genuine biological diversity of MSI-H tumors, there are some debates regarding technical aspects of MSI status determinations [133–136]. Therefore, it is unclear to what extent the data obtained on MSI-H cancers can be extrapolated to HNPCC-related tumors.

High antigenicity of MSI-H tumors makes them recognizable by the immune system. In order to overcome host defense mechanisms, MSI-H carcinomas express some immune checkpoint molecules thus creating local immunosuppressive environment [137]. Le et al. [138] investigated pembrolizumab (Keytruda, Merck), an antibody capable to inhibit PD1 pathway and thus restore peritumoral immune response, for the treatment of MSI-H cancers. This antibody induced objective tumor responses in all 6 patients with sporadic MSI-H tumors, but only in 3 out of 11 patients with the HNPCC syndrome; the reasons for these differences in efficacy in sporadic vs. hereditary MSI-H cancers remain obscure.

MSI-H tumors may have distinct spectrum of chemosensitivity, which is critically influenced by the individual pattern of somatically mutated genes [134, 136, 139]. There is an ongoing research aiming to develop synthetic lethal strategy for the targeting of MSI-H cancers [140].

There are studies aimed to develop systemic therapy for the familial adenomatous polyposis (FAP). Sulindac, a non-steroidal anti-inflammatory drug, was shown to be effective against colorectal adenomatous polyps. However, it has limited efficacy against duodenal carcinogenesis; duodenal carcinomas are the main cause of death in those FAP patients, who have already undergone colectomy. Synergistic interaction between COX2 and EGFR inhibitors was observed in murine FAP models. Combination of sulindac with erlotinib was assessed in a clinical trial involving 46 FAP patients in the experimental group and 46 subjects treated by placebo. Use of the above drugs resulted in clinically significant reduction of the number and size of duodenal polyps [141].

### Novel targeted agents for therapy of rare hereditary cancers

Medullary thyroid cancer is often driven by inherited activating mutation in RET oncogene. There are RET kinase inhibitors, vandetanib (Caprelsa, AstraZeneca) and cabozantinib (Cometriq, Exelixis), which demonstrate clinical activity against both hereditary and sporadic medullary thyroid carcinomas [142–145].

Everolimus (Afinitor, Novartis), an inhibitor of mTOR kinase, has been approved for the treatment of several tumor types. mTOR pathway is specifically activated in tumors arising in patients with tuberous sclerosis (TSC). Everolimus was systematically evaluated in TSC-related giant-cell astrocytomas and angiomyolipomas, and showed remarkable efficacy in these malignancies [146–148].

Vismodegib (Erivedge, Roche/Genentech) inhibits Hedgehog pathway via interaction with SMO protein. Its antitumor activity was examined in patients with basal-cell nevus (Gorlin) syndrome [149]. This drug reduced the size of existing basal-cell carcinomas and prevented the appearance of new lesions; however, 14 out of 26 subjects decided to discontinue the treatment due to adverse events. Vismodegib also showed some activity against keratocystic odontogenic tumors arising in patients with Gorlin syndrome [150]. There is evidence that this hereditary tumor disease may somehow be controlled with some other drugs. For example, a COX2 inhibitor, celecoxib slowed the increase of tumor burden in the treated patients as compared to placebo control [151]. Well-known triazol antifungal drugs (itraconazole, ketoconazole, posaconazole, etc.) turned out to have substantial inhibitory activity against Hedgehog pathway [152, 153]. They are capable to induce regression of basal cell carcinomas at least in a subset of patients [154, 155].

## Conclusions and perspectives

The number of known hereditary cancer syndromes will rapidly grow within next several years, thanks to the invention of whole exome sequencing [131]. Many of already identified hereditary cancer types are represented by exceptionally rare instances of the disease, and future investigations are likely to reveal even more uncommon cancer syndromes. In addition, unlike many other medical conditions, genetic diseases are often population-specific, i.e. their spread is limited by a few ethnic groups. It is unrealistic to expect that each hereditary cancer type will be subjected to systematic laboratory investigations and comprehensive clinical trials. There are some approaches which may facilitate search for novel treatment strategies for orphan and/or hereditary cancer types. For instance, collection and analysis of biological material from these patients deserve to be encouraged. In addition, some ex vivo testing for tumor drug sensitivity may turn out to be particularly practical in this clinical setting [156]. There are also some bioinformatic tools pretending to predict drug sensitivity of the tumor based on its molecular characteristics [157]. Finally, several potentially practice-changing investigations in this field became possible due to availability of large clinical databases [16, 27, 120]. Well-designed retrospective studies may help to significantly improve the use of existing cancer therapies.

## Abbreviations

aCGH, array comparative genomic hybridization; BC, breast cancer; EMEA, European Medicines Agency; FAP, familial adenomatous polyposis; FDA, Food and Drug Administration; HNPCC, hereditary non-polyposis colorectal cancer; HR, homologous recombination; LOH, loss of heterozygosity; MSI-H, high-level microsatellite instability; OC, ovarian cancer; PARP, poly(ADP-ribose) polymerase; PARPi, PARP inhibitors; pCR, pathologic complete response; PFS, progression-free survival; PLD, pegylated liposomal doxorubicin; TSC, tuberous sclerosis
